# Genome-wide mapping of ORC and Mcm2p binding sites on tiling arrays and identification of essential ARS consensus sequences in *S. cerevisiae*

**DOI:** 10.1186/1471-2164-7-276

**Published:** 2006-10-26

**Authors:** Weihong Xu, Jennifer G Aparicio, Oscar M Aparicio, Simon Tavaré

**Affiliations:** 1 Molecular and Computational Biology Program, University of Southern California, Los Angeles, CA, USA

## Abstract

**Background:**

Eukaryotic replication origins exhibit different initiation efficiencies and activation times within S-phase. Although local chromatin structure and function influences origin activity, the exact mechanisms remain poorly understood. A key to understanding the exact features of chromatin that impinge on replication origin function is to define the precise locations of the DNA sequences that control origin function. In *S. cerevisiae*, Autonomously Replicating Sequences (ARSs) contain a consensus sequence (ACS) that binds the Origin Recognition Complex (ORC) and is essential for origin function. However, an ACS is not sufficient for origin function and the majority of ACS matches do not function as ORC binding sites, complicating the specific identification of these sites.

**Results:**

To identify essential origin sequences genome-wide, we utilized a tiled oligonucleotide array (NimbleGen) to map the ORC and Mcm2p binding sites at high resolution. These binding sites define a set of potential Autonomously Replicating Sequences (ARSs), which we term nimARSs. The nimARS set comprises 529 ORC and/or Mcm2p binding sites, which includes 95% of known ARSs, and experimental verification demonstrates that 94% are functional. The resolution of the analysis facilitated identification of potential ACSs (nimACSs) within 370 nimARSs. Cross-validation shows that the nimACS predictions include 58% of known ACSs, and experimental verification indicates that 82% are essential for ARS activity.

**Conclusion:**

These findings provide the most comprehensive, accurate, and detailed mapping of ORC binding sites to date, adding to the emerging picture of the chromatin organization of the budding yeast genome.

## Background

Eukaryotic chromosomal DNA replication initiates from numerous loci, termed replication origins, distributed along each chromosome. The selection of chromosomal sites that will function as origins begins with the binding to DNA of the Origin Recognition Complex (ORC) [1,2]. During late M and early G1 phases, ORC, together with Cdc6 and Cdt1, directs the loading onto origin DNA of MCM complexes to assemble pre-replicative complexes (pre-RCs). Upon S-phase entry, activation of pre-RCs leads to DNA unwinding and the assembly of replisomes that carry out DNA synthesis [3,4]. Origins differ in their timing of activation during S-phase and their frequency of activation (maximum once per cell cycle). A clear understanding of factors that influence the efficiency and timing of initiation is lacking, although histone modification, nucleosome positioning, and transcription have been implicated [5-11]. Chromatin structure also appears to influence the selection of ORC binding sites [1,12,13].

In most eukaryotic cells, specific sequences do not appear to be required for ORC binding [13-15]. For example, in fission yeast, almost any highly A/T-rich sequence of sufficient length (~1 kb) can function as a replication origin. In *Xenopus *egg extracts and *Drosophila *embryos, apparently random, closely spaced DNA sequences serve as replication origins to facilitate rapid cell (nuclear) division cycles. During embryogenesis, the number of sites used as origins decreases with the onset of transcription. This correlation suggests that the establishment of chromatin domains related to transcription limits the number of ORC binding sites in the chromatin. In mammalian cells, transfection of almost any DNA fragment of sufficient length can support replication, suggesting that ORC binding is generally stochastic but requires the presence of an accessible region in the chromatin. Furthermore, the activity of certain sequences as replication origins in mammalian cells correlates with local differences in gene expression in different cell types or lineages [16-19].

*Saccharomyces cerevisiae *differs somewhat in that specific DNA loci that function as replication origins (termed Autonomously Replicating Sequences or ARSs) contain a consensus sequence (ARS Consensus Sequence or ACS) that is essential for ORC binding and origin function [1,20,21]. However, an ACS alone is not sufficient for origin function and this sequence is much more abundant than the number of ORC binding sites or functional replication origins [20,22]. In addition to an ACS, ARSs contain at least one A/T-rich region of DNA thought to act as a DNA unwinding element. Although an unwinding element is important for origin function, it is not required for ORC binding [23]. Thus, despite its sequence preference, it remains unclear exactly how ORC binding sites are selected from the many potential sequences; however, local chromatin structures and activities are probably important factors. Indeed, the great majority of origins locate to intergenic regions [24]. While this and other studies suggest active transcription and origin function are antagonistic [25,26], the effect of transcription factors on local chromatin can also be important. Detailed analysis of ARS1 suggests that factors that position nucleosomes surrounding origins (including ORC itself) influence origin initiation efficiency [5,27-29]. Additional information on the chromatin organization in relation to ORC at a variety of origins exhibiting different characteristics (e.g. timing, efficiency, chromosomal location) should yield valuable insights into the mechanisms that regulate origin function. A precise mapping of ORC binding sites throughout the genome is an important step in this direction.

The advent of DNA microarrays has enabled the genome-wide analysis of DNA replication dynamics and identification of replication origins in a number of eukaryotic organisms [30,31]. In *S. cerevisiae*, various approaches have been fruitful. Some studies have directly analyzed replication timing by monitoring the time at which specific DNA sequences double in copy number or incorporate a chemically distinct precursor (e.g. density-substitution) [32,33]. A very recent study mapped the presence of single-stranded DNA during replication, which is expected to identify sites undergoing DNA synthesis [34]. These types of studies have provided valuable data on the overall dynamics of genome duplication. These studies also identified the positions of ~300 active replication origins, typically to within several kilobases (4–10 kb).

An alternative approach to identifying replication origins used genome-wide location analysis to determine the positions of ORC and MCM proteins [24]. This study identified 429 sites predicted to have ARS function with a resolution of ~1 kb. This level of resolution facilitated experimental validation of the data set, demonstrating 79% positive predictive value (PPV, defined as the percentage of true sites among all called sites). Because this particular study analyzed the position of static protein complexes, it provided a more precise mapping than the timing-based studies, but did not by itself characterize the activity of the predicted sites. Thus, the replication dynamics and protein location analyses provide complementary information to help create an accurate description of genome duplication. These studies did not attempt to identify the exact DNA sequences (ACSs) bound by ORC, which are essential for origin function. However, one study has identified potential ACS using a purely sequence-based search algorithm (Oriscan) [22]. Among the top 350 Oriscan sites (which is similar to the total number of origins predicted or inferred by other studies), 56% matched known ARS or proARS sequences.

To provide a more accurate and complete map of essential origin sequences that bind ORC, we performed genome-wide location analysis of ORC and Mcm2p with a high-density, tiled oligonucleotide microarray. The 529 ORC and/or Mcm2p binding sites revealed potential ARSs with high accuracy. The resolution of this analysis allowed for precise localization of hundreds of functional ACSs, which serve as ORC binding sites, throughout the genome.

## Results

### Genome-wide identification of ORC and Mcm2p binding sites

Previously, we used genome-wide location analysis to map chromosomal binding sites of ORC and MCM proteins to about 1 kb resolution using DNA microarrays [21,24]. These microarrays contained about 12,000 cDNA probes, typically one for each open reading frame (ORF) and one for each intergenic region of the *S. cerevisiae *genome, with an average probe size of 618 bp. To map ORC and MCM binding sites with greater precision and facilitate the identification of ACSs within the identified binding regions, we performed genome-wide location analysis of ORC and Mcm2p using tiled oligonucleotide microarrays (NimbleGen) containing one 50mer oligonucleotide to represent each 80 bp segment of the genome, in triplicate. DNA enriched for ORC and Mcm2p binding sites was isolated by chromatin immunoprecipitation (ChIP) of ORC from M-phase cells and Mcm2p from G1-phase cells, respectively. Immunoprecipitated and total genomic DNA from each sample was differentially labeled and co-hybridized to the arrays.

**Figure 1:**
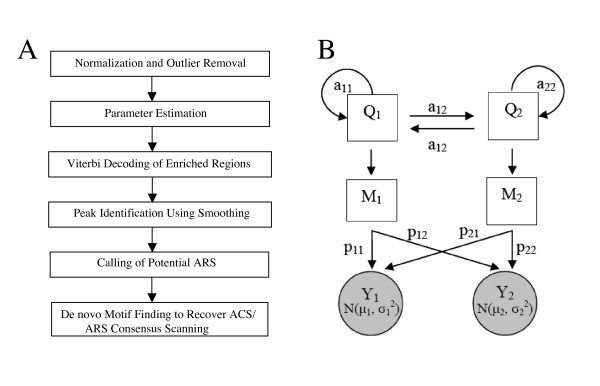
Analysis of ORC and Mcm2p whole genome localization experiments using a tiled oligonucleotide array. **A**. Strategy. **B. **The Hidden Markov Model with Mixture of Gaussian output. Round shapes represent continuous variables and square shapes represent discrete variables. Gray refers to observed variables.

Data were analyzed as outlined in Figure 1A (and see Methods). Briefly, a two-state Hidden Markov Model (HMM) with a mixture of Gaussian outputs was used to model the data (Figure 1B and see Additional file 1). This analysis identified 400 ORC-enriched regions and 634 MCM2-enriched regions. The ORC and MCM2 enriched regions intersect at 353 sites. Because the ORC and MCM2 intersecting regions generally do not overlap exactly, the union is used in defining a single site, resulting in 349 discrete (non-overlapping) regions that we refer to as ORC-MCM2 sites. The HMM-called regions, a total of 677 discrete sites, are divided into three groups: 47 ORC-only sites, 281 MCM2-only sites and 349 ORC-MCM2 sites (see Additional files 2, 3, and 4, respectively).

On these tiled oligonucleotide arrays, the immunoprecipitated target DNA (average shear size of ~1 kb) is expected to identify numerous probes for each binding site, with the probes' standardized log_2 _signal intensities (Z) forming a peak centered very close to the actual protein binding site. To locate the peak probe more accurately, the Z-values were smoothed using a moving average (see Methods). Within each HMM-called region, peaks of ORC and/or MCM2 signal were identified based on a continuous increase of the smoothed Z values (sZ) for at least five probes followed by a continuous decrease of sZ for at least five probes. A corresponding peak strength was calculated as the average Z value (avgZ) (see Additional files 2, 3 and 4).

### ARS prediction

We sought criteria to evaluate the merit of the three classes of binding sites for ARS prediction. The ORC-MCM2 class is anticipated to have the strongest predictive value because two different pre-RC proteins co-localize at these sites. Thus, we compared characteristics of the ORC-only and MCM2-only sites to the ORC-MCM2 sites. Among experimentally verified ARS sites, 95% are contained in the HMM-called data set (see Additional file 5); of these, 77% are defined by ORC-MCM2 sites, 23% by MCM2-only sites, and none by ORC-only sites. These results suggest that Mcm2p binding is a more sensitive predictor of ARS location than ORC binding.

**Figure 2:**
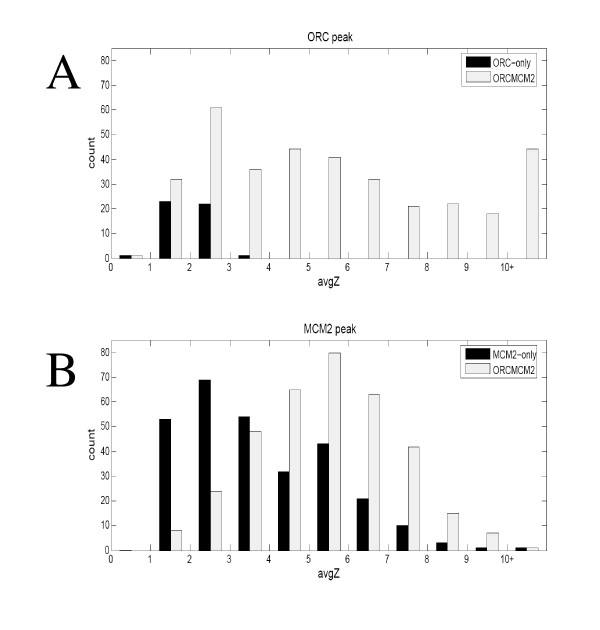
Peak strength in the three classes of binding sites. **A**. Comparison of ORC peak strength within ORC-only sites and ORC-MCM2 sites. **B**. Comparison of MCM2 peak strength within MCM2-only and ORC-MCM2 sites.

Examination of peak strength shows that true ARSs are associated with robust signals. For the 89 known ARSs identified in this analysis, 95% of the MCM2 peaks and 92% of the ORC peaks had avgZ ≥ 2.75. Comparison of signal strength between the three classes of sites shows that, on average, peaks of ORC-only sites are significantly weaker than peaks of MCM2-only sites as well as peaks of ORC or MCM2 in ORC-MCM2 sites (rank sum test p-value < 0.001 for all cases). Furthermore, peaks of MCM2-only sites are on average significantly weaker than peaks of ORC or MCM2 in ORC-MCM2 sites (rank sum test p-value < 0.001) (Figure 2). Combined with the proportions of known ARSs associated with each of these classes, these findings suggest that ORC-only sites have the lowest, ORC-MCM2 sites the strongest, and MCM2-only sites an intermediate predictive value.

**Figure 3:**
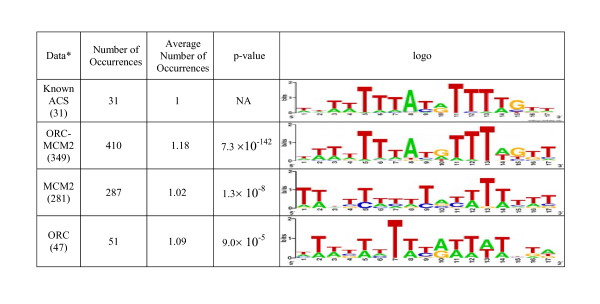
*De novo *motif finding within ORC-MCM2, MCM2-only and ORC-only sites. *Numbers in parentheses refer to the number of input sequences in each set. "Known ACS" denotes an alignment of 31 ACSs identified by mutation analysis and is provided for comparison. The sequence logo was generated using Weblogo [65].

A *bona fide *ARS is anticipated to contain an ACS that serves as the ORC binding site. An objective search for a common motif in each group of binding sites using *de novo *motif finding recovered the ACS motif from the ORC-MCM2 sites (Figure 3). In fact, the recovered motif is very similar to the extended ACS (EACS) described by Theis and Newlon [21], and also 89% similar to the motif generated by alignment of 31 previously identified, functional ACSs (see Additional file 12). For the MCM2-only group, a motif that is 70% similar to the EACS is recovered. No significant motif is recovered from the ORC-only sites (Figure 3). (See Methods for a description of the calculation of inter-motif similarity.) These results further support the idea that ORC-MCM2 sites are accurate predictors of origins whereas ORC-only sites appear to be poor predictors. The MCM2-only group probably contains a greater proportion of non-ARS sequences than the ORC-MCM2 group, complicating identification of the ACS motif in this group.

**Figure 4:**
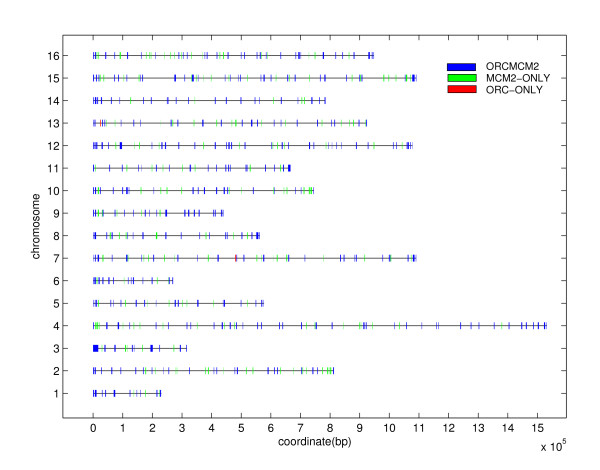
Distribution of nimARSs along the 16 *S. cerevisiae *chromosomes. Each horizontal line represents one chromosome. Blue bars represent nimARS sites identified by both ORC and MCM2; green bars represent nimARS sites identified by MCM2 only; and red bars represent nimARS sites identified by ORC only.

As ORC-MCM2 sites have a stronger average MCM2 peak signal than the average peak signal of the MCM2-only class, a threshold peak signal (avgZ ≥ 2.75) was established to select MCM2-only and ORC-only regions with a high probability of ARS activity. These were included in the set of potential ARSs called "nimARS". In total, 529 nimARS sites are defined, including 349 ORC-MCM2 sites, 178 MCM2-only sites, and two ORC-only sites (see Additional file 6). This data set includes 95% of experimentally confirmed ARSs (thus the sensitivity is 95%; sensitivity is defined as the percentage of sites that are called among all true sites in the genome). The chromosomal distribution of these sites is shown in Figure 4.

### Validation of ARS predictions and comparison with previous studies

Our analysis predicted 52 nimARS loci on chromosomes I and II. We tested the ARS function of 46 of these loci (five had been previously confirmed and one resisted analysis), and found that all but two have ARS activity (Table 1 and see Additional file 10). We also tested eight sites on chromosome X that were not predicted in the previous pro-ARS data set [24]. ARS activity was confirmed for six of these sites (3 strong, 2 weak and 1 very weak activity), one lacked activity, and one resisted analysis. On chromosomes III and VI, for which ARS activity has been comprehensively tested [35-37], the nimARS set predicts five new sites. Experimental analysis of these sites showed weak ARS activity for three sites, and two lacked activity (Table 1 and see Additional file 10). Comparing the cumulative experimental results from chromosomes I, II, III, VI, and X shows 94% PPV of the nimARS predictions (Table 1 and see Additional file 10) [24,35-37].

**Figure 5:**
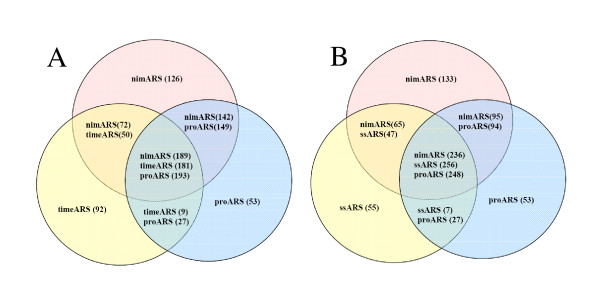
Venn diagrams showing overlap of data sets. **A. **nimARS, proARS [24] and timeARS [32]. **B. **nimARS, proARS and ssARS [34].

Comparison of our data with previous array-based origin predictions demonstrates considerable overlap. A Venn diagram shows the intersection of four data sets, proARS [24], timeARS [32], ssARS [34] and the current nimARS (Figures 5A and 5B). The criterion used to define corresponding sites is overlap between the defined regions. For the 332 timeARSs the region is defined as the 5 kb flanking each side of the peak. Of these timeARS regions, 231 (70%) intersect with 261 (49%) nimARS regions. For the 364 ssARS, the region is defined as the 4 kb flanking each side of the average position of ssDNA peaks at three time points. For this case, 301 (57%) nimARS regions intersect with 303 (83%) ssARS. The high overlap of nimARS with both timeARS and ssARS strongly suggests that nimARS includes the majority of active ARSs. Among the proARS sites, 342 (80%) overlap with 331 (63%) nimARS sites, numbers that closely correspond to the expected number of positives in the proARS data set (0.79 × 429 = 338). We tested 22 proARSs that do not overlap with a nimARS site and found that all 22 lack ARS activity (see Additional file 11). This finding underscores the greater accuracy of nimARS data. Also notable is the detection of ARS304 (MCM2-32), ARS319 (ORCMCM2-41) and ARS604 (ORCMCM2-105), three known ARSs that are inactive as chromosomal origins and have not been previously detected using array methodologies [24,32-35,37]. In fact, testing of 26 additional sites not identified by any previous array studies shows that 80% (21 of 26) have ARS activity, although this activity is frequently weak (see Additional file 10). The identification of numerous new ARSs indicates that the current analysis has higher sensitivity than previous studies, and includes some sites that have marginal activity.

### ORC and Mcm2p binding within nimARS regions

**Figure 6:**
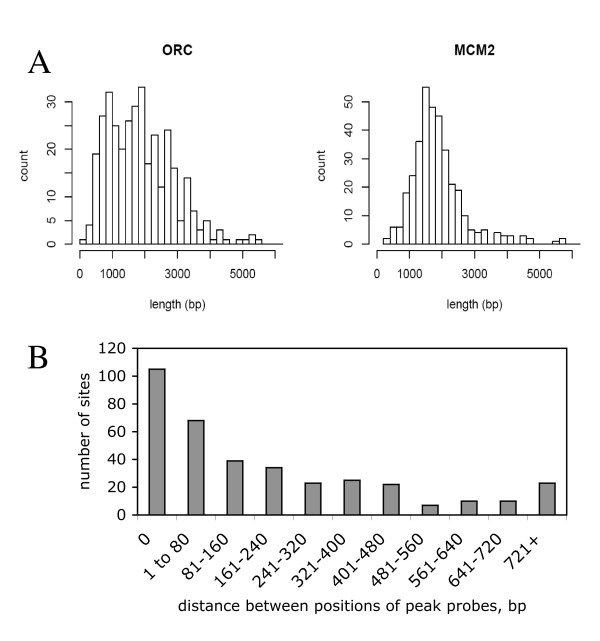
Comparison of ORC and MCM2 data within the set of ORC-MCM2 sites. **A. **Length of the ORC and MCM2 HMM-called regions is shown. **B. **Distance between the positions of probes that define ORC and MCM2 peaks in the ORC-MCM2 class of binding sites. Thus "0" means that the peaks identified are the same 50 bp probe and "721+" means that the peaks are more than 9 probes (spaced ~80 bp) apart.

Use of tiled oligonucleotide arrays yielded high-resolution data for which certain characteristics of ORC and Mcm2p binding *in vivo *as analyzed by ChIP could be examined. The mean lengths of individually defined ORC and MCM2 regions within the set of ORC-MCM2 sites are not significantly different (signed rank test of equality has p-value = 0.89), suggesting that the ORC and MCM complexes associate with similar lengths of chromatin (Figure 6A). To assess the relative positions of ORC and Mcm2p, we compared the distance between the ORC and MCM2 peaks within the ORC-MCM2 sites (Figure 6B). The peak of the ORC or MCM2 signal within each binding site is anticipated to identify the oligonucleotide probe closest to the protein-binding site. Although there was a significant range to the data, the most common occurrence was co-localization of the ORC and MCM2 peaks to the same probe, which represents an 80 bp region. These data are consistent with ORC and MCM proteins occupying similar locations within ARS chromatin.

### ACS identification

**Figure 7:**
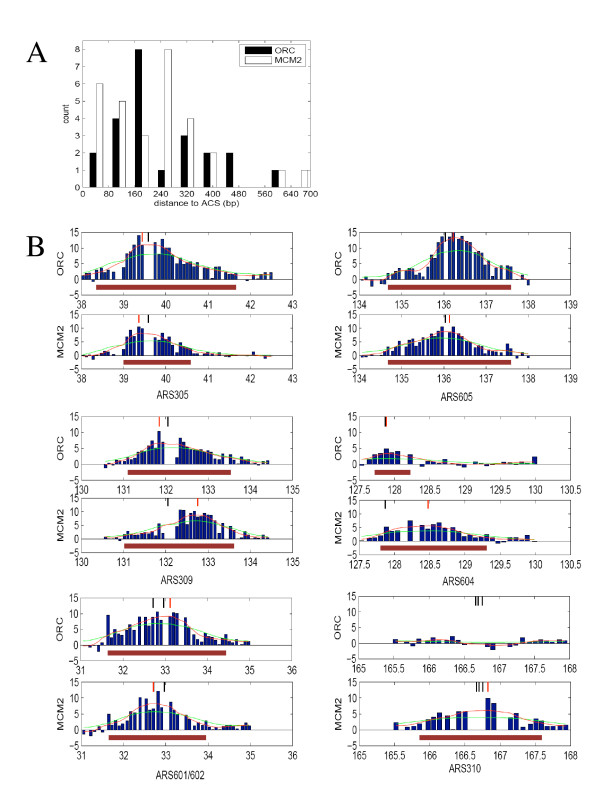
Known ACSs' locations relative to ORC and MCM2 peaks. **A. **Distance of known ACSs to closest ORC peak and MCM2 peak. **B. **Relative locations of ACS and ORC peak or MCM2 peak. The x-axis indicates the coordinates of probes on chromosomes in kilobases. The y-axis indicates signal strength by Z-value. The red tick attached to the top axis denotes the ORC or MCM2 peak and the black tick denotes the ACS location. The blue bars represent the Z-value at specified probe coordinates. The red and green lines indicate the smoothed Z-value (sZ) and average Z-value (avgZ) respectively. The horizontal red bar marks the regions called by HMM. For ARS305 and ARS605 both the ORC and MCM2 peaks are very close to the ACS location; for ARS309 and ARS604 the ORC peaks are located close to the ACS, but the MCM2 peaks are quite far due to shifted signal; ARS601/ARS602 and ARS310 have multiple proximate ACSs but do not confer multiple peaks in the data. Many ARSs show missing probes in the ACS region (15 out of 31 known ACSs have missing probes corresponding to the ACS).

Peak identification within the HMM-called regions provides a high-resolution map of ORC (and Mcm2p) binding that is expected to correspond to the location of an ACS. For the 31 ACSs that have been experimentally verified, ORC peaks are found on average 236 bp (95% confidence interval is 0 – 474 bp) from the defined ACS while MCM2 peaks average 222 bp (95% confidence interval is 0 – 525 bp) (Figure 7). A signed rank test shows no significant difference between the locations of the two distributions (p-value = 0.38). These distances are significantly shorter than the average shear size of the target DNA, suggesting that the shear size does not strictly limit the resolution on the tiling array due to presence of signal peaks in the data. The lack of a closer co-localization is at least partly due to the fact that probes corresponding to the exact ACS are frequently missing from the array due to the AT richness of these sequences (for examples, see Fig 7B).

The resolution of the nimARS data provides an opportunity to precisely define essential ACSs by narrowing a search to a relatively small region surrounding each nimARS data peak. A Positional Weight Matrix (PWM) generated from the 31 known ACSs yields a motif containing an EACS as well as three additional positions corresponding to the B1 element (of ARS1) (see Additional files 7 and 12). Interestingly, two of these three nucleotide positions had been previously mapped as sites of contact with ORC at ARS1 [38], suggesting this interaction is conserved. This EACS+B1 PWM was used to search a 1 kb window centered on each ORC and MCM2 peak. A 1 kb window was chosen because this roughly corresponds to the 95% confidence interval window for the distance of ORC and MCM peaks from known ACSs (see above). The EACS+B1 identified within the nimARS set are called nimACS (see Additional file 8). Using a p-value cutoff of 1.3 × 10^-4^, we identified 506 nimACS in 370 nimARS (78% have single ACS, 22% have multiple ACSs, see Additional file 8). (In comparison, this method and p-value identifies 3271 EACS+B1 sites within the entire genome.) The percentage of nimARS with multiple nimACSs is close to the proportion of known ARSs with multiple functional ACSs (5/25). A three-fold cross-validation (see Methods) shows that the nimACS includes 58% of known ACSs (thus the sensitivity is 58%). The accuracy of the defined nimACSs was tested by mutating 17 ACSs predicted within 14 ARSs on chromosome X (see Additional file 9). For 11 of these ARSs, mutation of the single predicted ACS eliminated ARS function. For the remaining three ARSs in which two ACSs were predicted in each, one of the two sites was essential for ARS function while the other was dispensable. These results indicate a PPV of 82%.

## Discussion

ARS identification in *S. cerevisiae *by genome-wide motif scanning has been hampered by the abundance of sequences with high similarity to the ACS, combined with the level of degeneracy of the ACS that supports function. Potential solutions to this problem include: (1) building larger motif models by including other concurrent motifs [39] or compositional information [22,40]; (2) assuming a specific motif distribution on chromosomes, e.g., a Hidden Markov Model [41,42]; and (3) narrowing down the regions to be searched. The first two methods rely on assumptions, which may introduce significant error. This study took the third approach, using a high-resolution array to map ORC and Mcm2p binding regions and confining the motif-search to this fraction (~5%) of the genome. A very recently published study took a fourth approach, analyzing phylogenetic conservation, in conjunction with motif searching and published microarray data to predict ACS locations [43].

We defined 529 nimARS loci throughout the *S. cerevisiae *genome that avidly bind ORC and/or Mcm2p. The vast majority of known ARSs (95%) are contained in the nimARS set and virtually all predicted sites exhibit ARS activity when tested (94%). Comparison to a recently determined set of chromosomally active replication origins (ssARS) shows that 83% are contained in the nimARS set [34]. Together, these analyses confirm the high accuracy of the nimARS data. The HMM analysis is capable of identifying even weak signals, while the target DNA identifies multiple probes on the tiled oligonucleotide array for each binding site, a redundancy that enhances accuracy. We further defined this data set by determining the signal peaks within the nimARS regions and constrained the motif search to a 1 kb segment centered on each peak. Within 370 (70%) of the nimARS loci we identified at least one nimACS, with an overall PPV of 82%.

Approximately one-third of the predicted nimARSs are loci where only Mcm2p was detected. Of the nimARSs for which ARS activity has been demonstrated (in this or previous studies), 34% (52/152; see Additional files 5 and 10) are MCM2-only sites. This observation suggests that the majority of these sites will prove to possess ARS activity. Furthermore, ORC binding was not detected at 23% of known ARSs, while nimACSs, which predict ORC binding, are found at 103 of 178 of the MCM2-only sites. Finally, we have no evidence (such as a unique motif) suggesting that MCM2-only sites represent a distinct function of Mcm2p, which might be independent of ORC.

As ORC is bound to chromatin throughout the cell cycle in budding yeast and is required to "load" the MCM complex onto DNA, the detection of many MCM2-only sites suggests that ORC is present but recalcitrant to detection by ChIP, perhaps due to local chromatin differences. Indeed, we analyzed ORC binding in G2/M-arrested cells because pre-RC assembly is thought to occlude detection of ORC in G1-arrested cells [44]. However, we have recently found that ORC binding at some ARSs is more strongly detectable by ChIP during G1- or S-phase (JGA and OMA, unpublished). One possibility is that Cdc6 stabilizes binding of ORC to weaker sites during G1 to permit MCM loading [45,46]. This would explain the loading of Mcm2p in G1-phase at sites where ORC failed detection in G2/M, and is consistent with the idea that ORC occupancy and stability varies at different sites depending on local chromatin features or DNA sequence variation.

Whereas ORC detection by ChIP may be context- or cell cycle-dependent, Mcm2p seems to be more reliably detected. This may reflect differences in the way the ORC and MCM complexes interact with DNA. In contrast to models of ORC-DNA binding along the A rich strand of DNA [38], the MCM complex is thought to encircle one or both strands of DNA [47,48]. Such a topology might enhance cross-linking of MCM to chromatin or otherwise stabilize these complexes for immunoprecipitation. A greater stability of the MCM complex in pre-RCs is supported by *in vitro *data in which high salt extraction of pre-RCs removes ORC (and Cdc6) from DNA, but not the MCM complex [49-52].

Significantly more pre-RCs are formed than are normally utilized to replicate the genome. This work predicts about 500 pre-RCs are formed while other studies indicate that about 260–360 of these are primarily responsible for replicating the genome [32-34]. Some inefficient pre-RCs retain potential for activation but fail to initiate replication because replication forks emanating from efficient, nearby origins replicate through these sites, thereby eliminating their activation potential (presumably by dismantling the pre-RC) [53,54]. However, some sites at which ORC and/or Mcm2p can be identified exhibit relatively weak initiation potential. In some cases weak initiation is due to local chromatin, such as at the mating-type silencer ARSs, because these ARSs function efficiently when removed from their normal chromatin context [55]. However, some ARSs function poorly in the plasmid context, suggesting that sequence variation results in reduced ORC binding or inefficient DNA unwinding [56]. Sequence variation explains the failure to identify a robust ACS (EACS+B1) within about 30% of the nimARS. Further study will be required to determine how the sequence composition of the ACS and the surrounding sequences, as well as the presence of nearby motifs bound by other DNA binding proteins, contribute to the differential efficiency of ARSs (although specific cases, such as the silencer-associated with ARSs, have been identified [9,57,58]).

The molecular evolution of sequence and activity among different ORC binding sites (and related sequences) occurs under different selective pressures than that of individual genes or unique sequences with defined functions, as indicated by lower levels of phylogenetic conservation of yeast origins compared to genes [43]. This is because most individual ORC binding sites likely contribute little or nothing to the organism's fitness. The main requirement is that a sufficient number of efficient origins be distributed along each chromosome to ensure rapid genome duplication. Hence, sequence changes that increase the origin efficiency of one ORC binding site may reduce selective pressure on ORC binding sites on the same chromosome (especially nearby), resulting in weaker binding sites or even sites with specialized function such as the silencers. Origin sequence evolution also may derive from selective pressures on local gene functions if these are influenced by the presence of ORC. Nevertheless, the presence of excess ORC binding sites can help ensure efficient genome duplication in case the normal origin initiation program is disrupted [59], and hence, the ability of ORC to bind sequence variants is functionally significant. The ability of ORC to bind varied DNA sequences appears to be particularly important in higher organisms where ORC binding appears to conform to differential chromatin contexts related to developmentally regulated gene expression.

## Conclusion

A central goal of current research in genomics is a precise and comprehensive mapping of all the protein-protein and protein-DNA associations that comprise the chromatin. Sequence-specific DNA-binding proteins such as ORC are thought to play an important role in establishing the local chromatin architecture by influencing the positioning (and possibly modifications) of histones, which bind DNA independently of sequence. Conversely, histones and other proteins likely influence ORC binding to DNA, although the relevant mechanisms remain obscure. In this study we used genome-wide location analysis to identify with high accuracy about 500 loci that bind ORC and/or Mcm2p proteins. Within ~70% of these sites we identified DNA sequences that match the consensus for ORC binding, and confirmed that about 80% were required for ARS function. Thus, we have defined the exact position of most ORC binding sites throughout the genome. These findings represents an important contribution that should facilitate future studies of how the interaction between ORC and other chromatin components influences replication origin function, as well as the possibility that ORC regulates chromatin structure or nuclear architecture.

## Methods

### Genome-wide location analysis

ORC and MCM2 binding sites were identified using genome wide location analysis [24,60]. Target DNA from strain OAy470 was obtained by chromatin immunoprecipitation (ChIP) as described [44]. ORC-bound DNA was isolated from cells arrested with nocodazole (10 μg/mL) for 3 hours at 23°C using anti-ORC polyclonal antibody (1:500) [61]. Mcm2p-bound DNA was isolated from cells arrested in G1 phase with 8.3 ng/mL α-factor (Sigma) for 4 hours at 23°C using anti-Mcm2p antibody (1:50, Santa Cruz). Immunoprecipitated DNA, as well as non-enriched total DNA, was amplified using ligation-mediated-PCR (LM-PCR). Enriched and total DNAs were end-labeled with Cy5 and Cy3, respectively, and co-hybridized to an array designed and synthesized by NimbleGen Systems, Inc. This array contained 124,991 50 bp oligonucleotides tiled every 80 bp across the *S. cerevisiae *genome, present in triplicate. DNA end-labeling, hybridization, and scanning were performed at NimbleGen Systems, Inc., which provided the final text file of foreground signal intensities.

### Normalization

The Cy5 and Cy3 foreground signals were converted to log ratio of enrichment defined as M = log_2_Cy5 - log_2_Cy3 and log intensity defined as A = (log_2_Cy5 + log_2_Cy3)/2 for each spot. Global loess normalization was applied to remove the systematic effect seen at low intensities on transformed data (see Additional file 13). The normalized M values within the three replicated blocks are highly correlated (ρ ≈ 0.74) for both ORC and Mcm2p. For each probe, the median of the three replicates was used for further analysis.

### HMM-MOG model

A Hidden Markov Model with Mixture of Gaussians (HMM-MOG) was used to fit the data (Figure 1B). The complete parameter set of the model can be described as λ = (π, A, B, P). Let Q_1 _represent the non-enriched state and Q_2 _represent the enriched state, π = (π_1_,π_2_) gives the initial probability of the two states. A = {a_11 _a_12_; a_21 _a_22_} denotes the transition probabilities between the two states: a_ij _is the probability of a transition from state Q_i _to state Q_j_. B={Y_1_, Y_2_} is the emission distribution, with Y_1_~N(μ_1_,σ_1_^2^) describing M values of non-enriched probes and Y_2_~N(μ_2_, σ_2_^2^) describing those of enriched probes. The emission distribution for each state is a mixture of these two Gaussians, but with different mixture proportions described by P = {p_11_, p_12_; p_21 _p_22_}. The idea is essentially to allow a proportion p_21 _of non-enriched probes in an enriched region and a proportion p_12 _of enriched probes in a non-enriched region.

The purpose of including the mixture is to overcome two typical types of error in a ChIP-chip experiment: (1) In non-enriched regions, some probes might behave similar to typical probes in enriched regions, due to possible cross-hybridization; this behavior will cause spikes for a small number of probes. (2) In enriched regions, some probes have weak signals comparable to typical probes in non-enriched regions, due to low hybridization efficiency, non-specificity, etc. These two types of error will occasionally cause improper transitions in a standard HMM (without the mixture) that result in false positive predictions or site breakage. Allowing some amount of false positive probes and some amount of false negative probes makes the HMM more robust to probe failures.

The HMM parameters can be given empirically or estimated from data using the well-known Baum-Welch algorithm for finding the maximum likelihood estimates (MLE) (cf. Rabiner 1989)[62]. To better estimate (π, A, B), we empirically set p_12_= 6% (false positive probes) and p_21 _= 1% (false negative probes). Experimental tests show that small changes in p_12 _and p_21 _do not change the results significantly. A Viterbi algorithm is used to decode the most probable state sequence to identify unique enriched regions. Because the parameters estimated from the data vary among different chromosomes (see Additional file 1), we standardized all M values to their corresponding Z values to facilitate further comparison.

### Peak identification

The Z values were smoothed using a three-probe window over six rounds, which corresponds to a weighted average of 13 probes (~1 kb, which corresponds to the average size of ChIP DNA fragments). The weight distribution is approximately 0.001 : 0.008 : 0.029: 0.069 : 0.123 : 0.173 : 0.193 : 0.173 : 0.123 : 0.069 : 0.029 : 0.008 : 0.001. A peak is defined by a continuous increase in the smoothed Z value (sZ) for at least five probes followed by a decrease of sZ for another five probes. For each enriched region we report only one peak (with the largest smoothed Z value) every 3 kb. This length was empirically chosen based on analysis of the length of peaks in enriched regions for known ARSs. For long HMM-defined regions (6% of total) multiple peaks were identified. The strength of each peak is defined as the average Z-value (avgZ) for 13 probes, covering ~1 kb. Each HMM region is denoted by the identifying protein(s) and a peak number (e.g. ORC-MCM2-34). If multiple peaks were identified in a region, an additional number is given (e.g. ORC-MCM2-33-1 and ORC-MCM2-33-2).

### Motif finding and building an EACS+B1 positional weight matrix

*De novo *motif finding was carried out separately on ORC-MCM2, ORC-only, and MCM2-only sites using BioProspector with the recommended significance level of p = 2.9 × 10^-7 ^(Z value = 5) [63]. A motif length of 17 bp was chosen based on the following prior information: (1) A *de novo *motif finding study on the pro-ARS data set [24] tested a range of motif lengths and showed that 17 bp is the optimum length for retrieving ACSs from the data [22,40]; (2) A previous study also described a 17 bp ACS motif [1,20,21]; (3) The alignment of 31 experimentally verified ACSs shows that these 17 bp are above the 95% quantile (0.17 bits) of the null distribution (estimated from 31 random sequences of 10 kb). Interestingly, the alignment also reveals that the 24^th^, 31^st^, 32^nd ^and 33^rd ^positions are significant, where the 32^nd ^and 33^rd ^positions had been previously mapped as B1 element contacted by ORC in ARS1 [38]. Thus we chose to form a two-block motif (named EACS+B1) composed of a 17 bp EACS followed by a 3 bp B1 exactly 13 bp apart (omitting 24^th ^position). A gapped PWM was built on the alignment. We used LOD score to measure the similarity of a test sequence to EACS+B1. Suppose the sequence we are examining is *a*_1_*a*_2_*...a*_33_. The likelihood of this sequence under the PWM, assuming it is an independent trials model, is

P(a1a2...a33)=f1,a1f2,a2...f17,a17f31,a31f32,a32f33,a33,
 MathType@MTEF@5@5@+=feaafiart1ev1aaatCvAUfKttLearuWrP9MDH5MBPbIqV92AaeXatLxBI9gBaebbnrfifHhDYfgasaacH8akY=wiFfYdH8Gipec8Eeeu0xXdbba9frFj0=OqFfea0dXdd9vqai=hGuQ8kuc9pgc9s8qqaq=dirpe0xb9q8qiLsFr0=vr0=vr0dc8meaabaqaciaacaGaaeqabaqabeGadaaakeaacqWGqbaucqGGOaakcqWGHbqydaWgaaWcbaGaeGymaedabeaakiabdggaHnaaBaaaleaacqaIYaGmaeqaaOGaeiOla4IaeiOla4IaeiOla4Iaemyyae2aaSbaaSqaaiabiodaZiabiodaZaqabaGccqGGPaqkcqGH9aqpcqWGMbGzdaWgaaWcbaGaeGymaeJaeiilaWIaemyyae2aaSbaaWqaaiabigdaXaqabaaaleqaaOGaemOzay2aaSbaaSqaaiabikdaYiabcYcaSiabdggaHnaaBaaameaacqaIYaGmaeqaaaWcbeaakiabc6caUiabc6caUiabc6caUiabdAgaMnaaBaaaleaacqaIXaqmcqaI3aWncqGGSaalcqWGHbqydaWgaaadbaGaeGymaeJaeG4naCdabeaaaSqabaGccqWGMbGzdaWgaaWcbaGaeG4mamJaeGymaeJaeiilaWIaemyyae2aaSbaaWqaaiabiodaZiabigdaXaqabaaaleqaaOGaemOzay2aaSbaaSqaaiabiodaZiabikdaYiabcYcaSiabdggaHnaaBaaameaacqaIZaWmcqaIYaGmaeqaaaWcbeaakiabdAgaMnaaBaaaleaacqaIZaWmcqaIZaWmcqGGSaalcqWGHbqydaWgaaadbaGaeG4mamJaeG4mamdabeaaaSqabaGccqGGSaalaaa@69A6@

where fi,ai
 MathType@MTEF@5@5@+=feaafiart1ev1aaatCvAUfKttLearuWrP9MDH5MBPbIqV92AaeXatLxBI9gBaebbnrfifHhDYfgasaacH8akY=wiFfYdH8Gipec8Eeeu0xXdbba9frFj0=OqFfea0dXdd9vqai=hGuQ8kuc9pgc9s8qqaq=dirpe0xb9q8qiLsFr0=vr0=vr0dc8meaabaqaciaacaGaaeqabaqabeGadaaakeaacqWGMbGzdaWgaaWcbaGaemyAaKMaeiilaWIaemyyae2aaSbaaWqaaiabdMgaPbqabaaaleqaaaaa@3346@ is the probability of observing base a_*i *_in position *i *in the PWM. The corresponding probability under the background model is

P(a1a2...a33)=qa1qa2...qa17qa31qa32qa33,
 MathType@MTEF@5@5@+=feaafiart1ev1aaatCvAUfKttLearuWrP9MDH5MBPbIqV92AaeXatLxBI9gBaebbnrfifHhDYfgasaacH8akY=wiFfYdH8Gipec8Eeeu0xXdbba9frFj0=OqFfea0dXdd9vqai=hGuQ8kuc9pgc9s8qqaq=dirpe0xb9q8qiLsFr0=vr0=vr0dc8meaabaqaciaacaGaaeqabaqabeGadaaakeaacqWGqbaucqGGOaakcqWGHbqydaWgaaWcbaGaeGymaedabeaakiabdggaHnaaBaaaleaacqaIYaGmaeqaaOGaeiOla4IaeiOla4IaeiOla4Iaemyyae2aaSbaaSqaaiabiodaZiabiodaZaqabaGccqGGPaqkcqGH9aqpcqWGXbqCdaWgaaWcbaGaemyyae2aaSbaaWqaaiabigdaXaqabaaaleqaaOGaemyCae3aaSbaaSqaaiabdggaHnaaBaaameaacqaIYaGmaeqaaaWcbeaakiabc6caUiabc6caUiabc6caUiabdghaXnaaBaaaleaacqWGHbqydaWgaaadbaGaeGymaeJaeG4naCdabeaaaSqabaGccqWGXbqCdaWgaaWcbaGaemyyae2aaSbaaWqaaiabiodaZiabigdaXaqabaaaleqaaOGaemyCae3aaSbaaSqaaiabdggaHnaaBaaameaacqaIZaWmcqaIYaGmaeqaaaWcbeaakiabdghaXnaaBaaaleaacqWGHbqydaWgaaadbaGaeG4mamJaeG4mamdabeaaaSqabaGccqGGSaalaaa@5B6A@

where qai
 MathType@MTEF@5@5@+=feaafiart1ev1aaatCvAUfKttLearuWrP9MDH5MBPbIqV92AaeXatLxBI9gBaebbnrfifHhDYfgasaacH8akY=wiFfYdH8Gipec8Eeeu0xXdbba9frFj0=OqFfea0dXdd9vqai=hGuQ8kuc9pgc9s8qqaq=dirpe0xb9q8qiLsFr0=vr0=vr0dc8meaabaqaciaacaGaaeqabaqabeGadaaakeaacqWGXbqCdaWgaaWcbaGaemyyae2aaSbaaWqaaiabdMgaPbqabaaaleqaaaaa@3121@ is the genomic frequency of base *a*_*i*_. The log-likelihood is defined as

LOD=∑i=1,2...17,31,32,33log⁡fi,aiqai.
 MathType@MTEF@5@5@+=feaafiart1ev1aaatCvAUfKttLearuWrP9MDH5MBPbIqV92AaeXatLxBI9gBaebbnrfifHhDYfgasaacH8akY=wiFfYdH8Gipec8Eeeu0xXdbba9frFj0=OqFfea0dXdd9vqai=hGuQ8kuc9pgc9s8qqaq=dirpe0xb9q8qiLsFr0=vr0=vr0dc8meaabaqaciaacaGaaeqabaqabeGadaaakeaacqWGmbatcqWGpbWtcqWGebarcqGH9aqpdaaeqbqaaiGbcYgaSjabc+gaVjabcEgaNnaalaaabaGaemOzay2aaSbaaSqaaiabdMgaPjabcYcaSiabdggaHnaaBaaameaacqWGPbqAaeqaaaWcbeaaaOqaaiabdghaXnaaBaaaleaacqWGHbqydaWgaaadbaGaemyAaKgabeaaaSqabaaaaaqaaiabdMgaPjabg2da9iabigdaXiabcYcaSiabikdaYiabc6caUiabc6caUiabc6caUiabigdaXiabiEda3iabcYcaSiabiodaZiabigdaXiabcYcaSiabiodaZiabikdaYiabcYcaSiabiodaZiabiodaZaqab0GaeyyeIuoakiabc6caUaaa@5565@

The LOD score was converted to a p-value based on the null distribution generated by scanning EACS+B1 throughout chromosome VI, excluding all identified ARSs. Sensitivity was obtained by a three-fold cross-validation. Briefly, the 31 ACSs were divided randomly into subgroups of 10, 10, and 11 ACSs, and each subgroup was scored by the PWM with parameters estimated from the other two subgroups. The number of ACSs with scores above a chosen threshold divided by 31 indicates the sensitivity.

The inter motif similarity between motif A and B is defined as

S(A,B)=1−12W∑i=1W∑j=14|fi,jA−fi,jB|,
 MathType@MTEF@5@5@+=feaafiart1ev1aaatCvAUfKttLearuWrP9MDH5MBPbIqV92AaeXatLxBI9gBaebbnrfifHhDYfgasaacH8akY=wiFfYdH8Gipec8Eeeu0xXdbba9frFj0=OqFfea0dXdd9vqai=hGuQ8kuc9pgc9s8qqaq=dirpe0xb9q8qiLsFr0=vr0=vr0dc8meaabaqaciaacaGaaeqabaqabeGadaaakeaacqWGtbWucqGGOaakcqWGbbqqcqGGSaalcqWGcbGqcqGGPaqkcqGH9aqpcqaIXaqmcqGHsisldaWcaaqaaiabigdaXaqaaiabikdaYiabdEfaxbaadaaeWbqaamaaqahabaWaaqWaaeaacqWGMbGzdaqhaaWcbaGaemyAaKMaeiilaWIaemOAaOgabaGaemyqaeeaaOGaeyOeI0IaemOzay2aa0baaSqaaiabdMgaPjabcYcaSiabdQgaQbqaaiabdkeacbaaaOGaay5bSlaawIa7aaWcbaGaemOAaOMaeyypa0JaeGymaedabaGaeGinaqdaniabggHiLdaaleaacqWGPbqAcqGH9aqpcqaIXaqmaeaacqWGxbWva0GaeyyeIuoakiabcYcaSaaa@573F@

where *W *is the motif length,fi,jA
 MathType@MTEF@5@5@+=feaafiart1ev1aaatCvAUfKttLearuWrP9MDH5MBPbIqV92AaeXatLxBI9gBaebbnrfifHhDYfgasaacH8akY=wiFfYdH8Gipec8Eeeu0xXdbba9frFj0=OqFfea0dXdd9vqai=hGuQ8kuc9pgc9s8qqaq=dirpe0xb9q8qiLsFr0=vr0=vr0dc8meaabaqaciaacaGaaeqabaqabeGadaaakeaacqWGMbGzdaqhaaWcbaGaemyAaKMaeiilaWIaemOAaOgabaGaemyqaeeaaaaa@32D1@ and fi,jB
 MathType@MTEF@5@5@+=feaafiart1ev1aaatCvAUfKttLearuWrP9MDH5MBPbIqV92AaeXatLxBI9gBaebbnrfifHhDYfgasaacH8akY=wiFfYdH8Gipec8Eeeu0xXdbba9frFj0=OqFfea0dXdd9vqai=hGuQ8kuc9pgc9s8qqaq=dirpe0xb9q8qiLsFr0=vr0=vr0dc8meaabaqaciaacaGaaeqabaqabeGadaaakeaacqWGMbGzdaqhaaWcbaGaemyAaKMaeiilaWIaemOAaOgabaGaemOqaieaaaaa@32D3@ are the observed frequency of base j at position i in motif A and B respectively. The similarity is between 0 and 1; multiplying by 100 gives the similarity as a percentage.

### Determination of ARS and ACS function

ARS activity was determined by testing the ability of a sequence of interest to confer replication to a plasmid otherwise lacking a functional yeast ARS as described by Wyrick et al. [24], or by a co-transformation approach that takes advantage of yeast's high frequency of homologous recombination. For the latter method, the sequence of interest was amplified from yeast genomic DNA using primers that each contains 20 bp of homology to the sequence of interest and 40 bp of homology to either end of a gapped *CEN4/URA3 *vector lacking an ARS. The amplified product was co-transformed into yeast (*ura3*-*1*) with the gapped vector, and transformants were selected on -URA. A high frequency of transformation depended on the presence of an ARS sequence in the amplified DNA, while colony size reflected the efficiency of ARS function. For classification, ARS305 was used as the standard for normal ARS function and ARS604 as representative of a weak ARS. Weak ARSs exhibit a high frequency of transformation, but form smaller colonies than cells harboring ARS305, requiring about three days, rather than two, to form a colony ~3 mm in diameter. Very weak ARSs also showed a high frequency of transformation, but colonies were small after three days and grew slowly upon restreaking. These assays were performed in duplicate.

To test the requirement of potential ACSs for ARS function, PCR primers were designed to amplify the ARS region in two fragments each of which has an endpoint in the 11 bp ACS. The ACS was replaced with a restriction site to allow ligation of the two fragments. The distal ends of these two fragments also contained introduced restriction sites for ligation into a vector lacking yeast ARS function. If deletion of an ACS resulted in loss of the high transformation frequency of the ARS, the ACS was denoted as functional.

### Data deposition

Data from this work is being submitted to the *Saccharomyces *Genome Database. Data will also be available at the DNA Replication Origin Database [64], which includes a graphic viewer format of the nimARS data similar to that of Figure 7B.

## Authors' contributions

WX performed computational analyses. JGA performed experimental work. All authors contributed to the conception and experimental design, and to manuscript drafts and revisions.

## Supplementary Material

Additional file 1 HMM parameters. See Methods for explanation. Click here for file

Additional file 2 ORC-only sites. Click here for file

Additional file 3 MCM2-only sites. Click here for file

Additional file 4 ORC-MCM2 sites. Click here for file

Additional file 5 Identification of known ARS. Click here for file

Additional file 12 Logo of 31 known ACSs demonstrates the EACS+B1 element. The dashed line shows 95% quantile (0.17 bits) of information content distribution of 31 10,000 bp random sequences. Based on the cutoff, we used a 17 bp EACS + 3 bp B1 to construct a gapped PWM to scan nimARS. Click here for file

Additional file 6 Set of nimARS. Click here for file

Additional file 10 Verified nimARS. Coordinates of regions tested for ARS activity are provided. ARS names are assigned to be consistent with Wyrick et al. [24] and the DNA replication origin database [64]. Click here for file

Additional file 11 False predictions of Wyrick et al. [24]. Click here for file

Additional file 7 Known ACS. Click here for file

Additional file 8 Predicted nimACS. Click here for file

Additional file 9 Verified nimACS. Click here for file

Additional file 13 Normalization using global loess. The red line indicates the loess line. M is the log of ratio of IP divided by total (also termed enrichment score) and A is the average log intensity. Click here for file

## Figures and Tables

**Table 1:** Summary of ARS testing results and known ARSs for nimARSs on five chromosomes.

*Chromosome*	*I*	*II*	*III*	*VI*	*X*	*Total*
Known ARS	2	3	13	13	22	53
ARS activity	11	33	0	3	6	53
No ARS activity	0	2	1	1	3	7
Untested	0	1	0	0	1	2
Total nimARS	13	39	14	17	32	115
