# Supplementary figures and images for: Genome-wide mapping of ORC and Mcm2p binding sites on tiling arrays and identification of essential ARS consensus sequences in S. cerevisiae
Source: BMC Genomics. 2006 Oct 26;7:276. doi: 10.1186/1471-2164-7-276 (PMC1657020; doi:10.1186/1471-2164-7-276)

EACS

B1

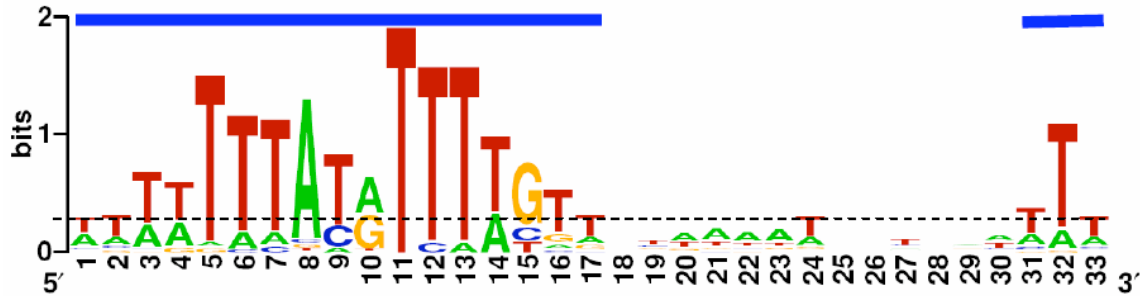

Supplement: Additional file 12 — Logo of 31 known ACSs demonstrates the EACS+B1 element. The dashed line shows 95% quantile (0.17 bits) of information content distribution of 31 10,000 bp random sequences. Based on the cutoff, we used a 17 bp EACS + 3 bp B1 to construct a gapped PWM to scan nimARS. [file 1471-2164-7-276-S12.pdf]

**ORC,Raw**

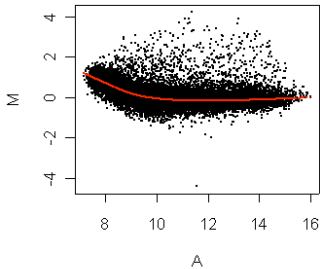

**MCM2,Raw**

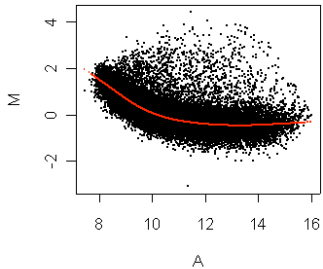

**ORC,Global Loess Normalized**

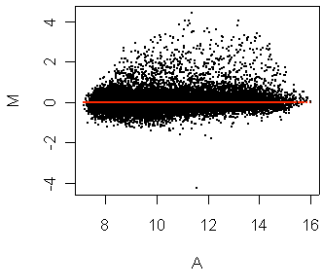

**MCM2,Global Loess Normalized**

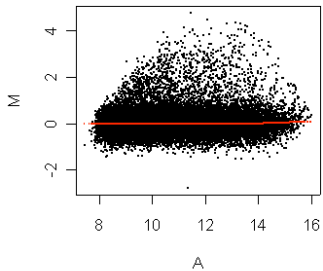

Supplement: Additional file 13 — Normalization using global loess. The red line indicates the loess line. M is the log of ratio of IP divided by total (also termed enrichment score) and A is the average log intensity. [file 1471-2164-7-276-S13.pdf]
