# Supplementary material for: Genome-wide mapping of ORC and Mcm2p binding sites on tiling arrays and identification of essential ARS consensus sequences in S. cerevisiae
Source: BMC Genomics. 2006 Oct 26;7:276. doi: 10.1186/1471-2164-7-276 (PMC1657020; doi:10.1186/1471-2164-7-276)
Supplement: Additional file 9 — Verified nimACS. [file 1471-2164-7-276-S9.doc]

| ID | Start | End | Np | ORC | | MCM2 | | EACS+B1 | | | | | |
| --- | --- | --- | --- | --- | --- | --- | --- | --- | --- | --- | --- | --- | --- |
|  |  |  |  | Pos | avgZ | Pos | avgZ | Pos | Score | Pvalue | St | Pattern | Exp |
| ORCMCM2-173 | 25 | 2381 | 29 | 312 | 10.6 | 312 | 18 | 268 | 6.0 | 2.3E-6 | f | TTATTTTATGTTTACTTTTTATAGACTGTCTTT | + |
| ORCMCM2-174 | 6470 | 9729 | 30 | 7776 | 13.4 | 7776 | 7.3 | 7732 | 5.8 | 2.3E-6 | r | TATTTTTATGTTTAGGTGATTTTGGTGGTGATT | + |
| MCM2-133-1 | 15779 | 19046 | 37 | NA | NA | 16769 | 7.0 | 16466 | 3.2 | 9.7E-5 | f | TTTTTTTATTTTTATGTATGAGAACTGCCGAAA | + |
| ORCMCM2-175 | 23258 | 25477 | 24 | 23674 | 4.4 | 23594 | 6.5 | 23920 | 3.9 | 3.8E-5 | f | AAATTTTTAGTTTTGTTATAATAAACGACTTTT | + |
|  |  |  |  |  |  |  |  | 23819 | 3.4 | 7.0E-5 | f | CTTTTTAAAATTTTGTTTATACTCAATTTCGTC | - |
| ORCMCM2-176 | 66736 | 68872 | 24 | 67465 | 5.6 | 67859 | 4.9 | 67663 | 3.8 | 3.8E-5 | f | AAATTATATGTTTTTGTCCTGGGCGCGACATTC | + |
|  |  |  |  |  |  |  |  | 67831 | 3.5 | 5.6E-5 | r | TTTTTTTCTGTTTTTCTTCCTATCTTGTATTTG | - |
| ORCMCM2-177 | 98352 | 100955 | 29 | 99941 | 5.8 | 99264 | 4.6 | 99618 | 7.0 | 2.3E-6 | r | TTTATTTATGTTTTGTTTAACTGACGGTGTTTT |  |
|  |  |  |  |  |  |  |  | 99492 | 4.3 | 1.4E-5 | f | TTTTTTAATTTTTTTTTTTTAATTTTTTTTTTT |  |
|  |  |  |  |  |  |  |  | 99751 | 3.5 | 5.6E-5 | r | TGGTATTATATTTTGGCCATATTCACTGCCTTT |  |
|  |  |  |  |  |  |  |  | 99667 | 3.1 | 1.1E-4 | r | ATCATTTAAATTTTGATTTGACACTTTGATTTG |  |
| ORCMCM2-178 | 111895 | 116320 | 47 | 113335 | 6.3 | 113335 | 4.9 | 113663 | 4.2 | 2.5E-5 | f | TTTTTTTACTTTTACCATTTTCTGTAAGAATTT | - |
|  |  |  |  |  |  |  |  | 113745 | 3.6 | 5.4E-5 | r | TAAATATATATTTAGTTATGGAAATTCAATAAA | + |
| MCM2-135 | 160725 | 162685 | 20 | NA | NA | 161785 | 7.4 | 161660 | 5.1 | 2.3E-6 | f | TATATCTATGTTTAGTATACCCAAAGGGTATTT | + |
| ORCMCM2-180 | 204280 | 205355 | 13 | 204755 | 9.4 | 204755 | 4.6 | 204869 | 4.4 | 1.4E-5 | f | TTTTTTTATTTTTTTTGGCTCATCTACGTAATC |  |
|  |  |  |  |  |  |  |  | 204910 | 3.3 | 8.5E-5 | f | TTTTATTAAATTTTGAAGCTATGAGTTTCTTTC |  |
| MCM2-137 | 227635 | 229405 | 15 | NA | NA | 229085 | 2.9 | 228795 | 3.7 | 5.2E-5 | r | AATATTTATATTTATGTACAGTTTTACATTGTA | + |
| MCM2-138 | 248405 | 250395 | 21 | NA | NA | 249175 | 7.3 | 248898 | 4.1 | 2.5E-5 | r | ACTTTTTAAGTTTAGCGGAACAAAACTAACATT |  |
| ORCMCM2-181 | 335845 | 339105 | 36 | 337590 | 6.7 | 337590 | 5.3 | 337268 | 5.4 | 2.3E-6 | f | TTTTTTTATGTTTAGCTAAGTAAAAGCAGCTTG | + |
| ORCMCM2-184 | 415515 | 418595 | 29 | 417940 | 3.8 | 417495 | 4.9 | 417311 | 6.4 | 2.3E-6 | r | ATTTTTTATATTTAGTTTACTTTCCAGTCAATT | + |
| ORCMCM2-185 | 441035 | 444475 | 36 | 443045 | 8.6 | 442365 | 4.7 | 442645 | 5.7 | 2.3E-6 | r | TAAATTTATATTTTGTTCGTAAAAAGAAAAATT | + |
| ORCMCM2-187 | 539670 | 541745 | 20 | 540355 | 4.7 | 540355 | 5.8 | 540522 | 5.3 | 2.3E-6 | f | AATTTTTACATTTAGTAATATCTAATAACATAT | + |
| MCM2-145 | 608675 | 610065 | 14 | NA | NA | 609355 | 4.3 | 609675 | 3.0 | 1.3E-4 | r | CTTTGTTATGTTATCTTACGGCTAAAACTAATA |  |
| ORCMCM2-188 | 612235 | 613645 | 15 | 612725 | 5.6 | 613185 | 5.3 | 612993 | 3.4 | 7.9E-5 | r | TTTTTTTATCTTTAGTTGCCTAAATACTATTTA | + |
| MCM2-146 | 643815 | 644765 | 8 | NA | NA | 644155 | 4.9 | 644324 | 3.9 | 3.8E-5 | f | ATTGTTCAAGTTTTGTATTGATTTTAAGTAATC |  |
| MCM2-147 | 653440 | 655430 | 20 | NA | NA | 654385 | 5.9 | 654465 | 6.1 | 2.3E-6 | r | TTTATTTACATTTTGGTCATTTGAAAATACTTT | + |
| ORCMCM2-189 | 683165 | 684845 | 18 | 683705 | 7.1 | 683785 | 4.3 | 683736 | 4.2 | 1.8E-5 | f | ATTTTTCGTATTTAGTGATTATAATACTTATTA |  |
| MCM2-150 | 728515 | 731275 | 30 | NA | NA | 729820 | 7.4 | 730033 | 5.5 | 2.3E-6 | f | ATTTTTTACGTTTTCTCCATATTTCGAATTGTT |  |
|  |  |  |  |  |  |  |  | 729618 | 4.52 | 1.1E-5 | f | TTTTTATATGTTTTGTTTGCTGTCGTCACAAGA |  |
|  |  |  |  |  |  |  |  | 730091 | 3.5 | 5.6E-5 | r | TTTTTTATTATTTTGATTGAAGAAGGAACAATT |  |
| MCM2-151-2 | 734370 | 738265 | 40 | NA | NA | 737435 | 5.9 | 737133 | 3.37 | 7.9E-5 | r | ACTATTTAAATTTTTATTTTTCGGTGTTATTTT |  |
| ORCMCM2-191 | 743975 | 745615 | 17 | 745025 | 11.6 | 745105 | 5.2 | 744852 | 5.77 | 2.3E-6 | f | TATTTTTATGTTTAGGTGATTTTAGTGGTGATT |  |
